# Risks and Opportunities of RegTech and SupTech Developments

**DOI:** 10.3389/frai.2019.00014

**Published:** 2019-07-30

**Authors:** Giorgio Gasparri

**Affiliations:** ^1^Commissione Nazionale per le Società e la Borsa, Rome, Italy; ^2^Financial Markets Law, University of Naples Federico II, Naples, Italy

**Keywords:** RegTech, SupTech, FinTech, artificial intelligence & law, algorithmic process, knowledge impairment, equality of arms, transparency & disclosure

In my short remarks I would like to focus on a few key-points relating to the opportunities and vulnerabilities associated with the implementation of new technologies in the financial sector, with particular regard to the RegTech topic—that implies the deployment and regulation of information technologies used in the context of regulatory compliance, including tasks such as regulatory reporting, securities transaction monitoring, and risk management—and the SupTech topic, related to the technologies used by supervisory authorities^1^.

As everyone knows, what we intend as “FinTech” is the abbreviation for “Financial Technology,” namely the nowadays ubiquitous application of technology to the delivery of financing, payment, investment, and consulting services, which has become a powerful driver of innovation in the financial services market^2^.

Among the main trends, we can identify key areas of application, including payments, personal finance, lending, investments, banking, and the new developments in robo-advisory. These new services offer the advantage of being “on-the-go,” efficient, easily accessible and convenient.

Relevant developments are also taking place in relation to applications of distributed ledger technology (DLT), artificial intelligence (AI)^3^, Machine Learning techniques^4^, Big Data Analytics^5^, RegTech^6^, and SupTech^7^, precisely.

In particular, it should be borne in mind that after an initial phase in which most regulators have chosen to observe, sometimes closely, the potential of technology start-ups (a sort of RegTech 1.0), it is high time that start-ups work alongside regulators in meeting challenges (that is the emergence of Regtech 2.0).

The transformational potential of RegTech has been confirmed in recent years with investments that more than tripled from $1.2 billion in 2017 to $3.7 billion in 2018^8^.

Having regard to these transformations and huge investments, we can assume that RegTech will not only provide significant efficiency gains for compliance and reporting functions: it will strongly change market structure and supervision^9^.

We can say that, at the moment, the widespread adoption of RegTech/SupTech solutions certainly seems to reduce certain risks: for example, the use of machine learning tools to monitor potential market abuse practices probably has the potential to improve market integrity; authorities such as the ECB and the U.S. Fed are using Natural Language Processing (a form of AI) to help them identify financial stability risks.

Another potential application of AI and Machine Learning is to detect collusive behavior and price manipulation in the securities market—potential misconducts that can be especially hard to detect using traditional methods^10^.

Compared with the high false-positive detection rate of traditional surveillance systems, based on human skill and knowledge, “Machine Learning-based” surveillance systems—through mathematical optimization techniques—have been able to reduce “false alarm” rates.

Some regulators are also employing technological tools to reduce the need for humans to manually conduct tricky network-analysis. This approach involves analyzing years of raw “order book data” with modern network-analysis techniques. The benefit of this system is not only the processing of large volumes of data, but also the detection of complicated network relationships across long time periods and often involving huge numbers of participants.

Semi-supervised Machine Learning algorithms can handle certain cases for which human experts' judgement has traditionally been necessary. In particular, Natural Language Processing technology could be used to automatically analyse many years of financial transaction data and extract meaningful information on which Machine Learning algorithms can profitably operate.

However, further improvement and refinement of these Machine Learning-based systems is needed, due to the lack of case-based training.

Other challenges include how Machine Learning can be used to detect previously unknown misconduct and how the results from the Machine Learning algorithms can be interpreted.

In the end, the increasing adoption of AI and Big Data can help investment firms and issuers of financial instruments to be more efficient and therefore may lead to cost reductions for investors, but—as the phenomenon is still evolving—operational risks are present^11^.

After all, the “self-supporting” market penetration process immanent to Big Data and AI can lead to the emergence of monopoly-like market structures. Dominant providers of Big Data and AI tools can then become of systematic importance for financial markets. As AI increases interconnectedness and as many investment firms use the same tools, there is an increased concentration risk and a higher vulnerability to single points of failure (SPOFs).

Likewise, AI may be used for SupTech tools: it could help us regulators to validate—and even analyse—a lot of (structured and unstructured) data. We could then become even faster in spotting new risks and dealing with them, but effectiveness depends on quality of underlying data, in terms of cleanliness and accessibility^12^.

And there are another risks that we must keep in mind: just think of the legal risks that could arise when we start to handle ever-larger amounts of sensitive datasets. We also need to monitor closely IT and cyber risks: we must find suitable ways to ensure the very high levels of resilience required. So, when we supervisors start to heavily apply digital tools, we ourselves must be as cautious as we ask investment firms to be.

One last issue recently discussed is that of the limits of the use of algorithms in the public decision-making mechanisms^13^. In particular, it is good to start thinking about whether decisions taken *only* on the basis of the elaboration of an algorithm and intended to affect the legal sphere of individuals are compatible with the traditional legal and, where present, constitutional guarantees^14^.

The risk of circumventing the principles of the law and, possibly, of the Constitution would be much more serious in the case of Deep Learning Algorithms. These Algorithms, being able to rework the rules on the basis of which they were programmed, could take decisions incomprehensible to the same supervisor: no one could ensure that the rules applied by the algorithms comply with the law and the regulator would give up its flexibility and discretion in favor of algorithms that feed themselves and define their own rules using a dangerous “black box approach”^15^…

Rules applied by dynamic AI could ultimately end up being impossible to determine in advance, with a consequent paradoxical violation of the principle of legal certainty which was precisely the aim pursued by the supporters of the use of predictive algorithms…

It therefore seems highly desirable that regulators be able to master the algorithmic process in order to explain to the concerned parties, in detail and in an intelligible form, that the decision was taken in accordance with the law.

With regard to law enforcement, consider, moreover, the problems posed by the evidence collected and generated in a fully automated way: algorithmic evidence introduces an extreme form of knowledge impairment, since the probative result may not be subject to criticism, because the inaccessibility of the source code or other characteristics of the software do not allow the party against whom the evidence is introduced into the proceeding to dispute its accuracy and reliability. This clearly poses a major problem of equality of arms.

Of course, the most immediate answer to the problem of the opacity of the algorithmic and computational processes is—as usual—more transparency. One can say: “Let's allow access to the source code, inputs and outputs of the software.” However, transparency may run the risk of subtly replacing the rule of law: in fact, open access to those data may not be useful, since only computer experts are able to draw meaningful and comprehensible elements from them^16^. So, transparency is necessary, but it is not enough^17^.

In addition, the data, collected or processed digitally, risk becoming reliable *in themselves*, because the verification of the process that generated them is too complex or escapes—at least in part—an ex post check, because of the use of more or less sophisticated forms of AI.

In this context, the authority could have access, for obvious reasons, to the best technologies, the results of which would be transferred to the legal proceeding as evidence. The concerned parties, on the other hand, might not have the possibility of convincingly questioning the reliability of such evidence, since they might not have the necessary elements for falsification.

The Courts, ultimately, in the case of re-examination of the decision, might have no reason to suspect the evidence, in the absence of concrete doubts adduced by the defense, relying dangerously on the belief that the digital data are free of risks of inaccuracy.

So, unfortunately—as I tried to point out—the problems are many and the debate, on these and other difficult and thorny issues, has just begun.

For sure, regulators and supervisors need to build new skills and new attitudes and the European Union must adopt a common and determined regulatory stance in these areas, at least in terms of the basic issues^18^.

## Author's Note

The speech was given by the author during “*Fin-Tech HO2020: RegTech workshop BigData Analytics*,” organized by Mode Finance on 29 March 2019 at the Milan Fintech District.

## Author Contributions

The author confirms being the sole contributor of this work and has approved it for publication.

### Conflict of Interest Statement

The author declares that the research was conducted in the absence of any commercial or financial relationships that could be construed as a potential conflict of interest.

## Footnotes

^1^See also Armstrong (2018), Enria (2019), ESMA (2019), and Karakas and Stamegna (2019).

^2^See Financial Stability Board (FSB) (2017a) and Carmona et al. (2018).

^3^On this topic see the Communication from the European Commission on *Artificial Intelligence for Europe*, COM (2018) 237 final (https://eur-lex.europa.eu/legal-content/EN/TXT/PDF/?uri=CELEX:52018DC0237&from=EN).

^4^On this topic see Internet Society, *Artificial Intelligence and Machine Learning: Policy Paper*, 2017 (https://www.internetsociety.org/wp-content/uploads/2017/08/ISOC-AI-Policy-Paper_2017-04-27_0.pdf).

^5^On this topic see the European Parliament resolution of 14 March 2017 on *Fundamental rights implications of big data: privacy, data protection, non-discrimination, security and law-enforcement* (2016/2225(INI)); *European Commission Financial Services User Group (FSUG), Assessment of current and future impact of Big Data on Financial Services*, June 2016, 10 (https://ec.europa.eu/info/file/46888/download_en?token=Mgdghb0P).

^6^Institute of International Finance (IIF), *RegTech in Financial Services: Technology Solutions for Compliance and Reporting*, March 2016, and *Regtech: exploring solutions for regulatory challenges*, Washington DC, October 2015.

^7^On this topic see European Supervisory Authorities (Esas) Joint Committee, *Final Report on Big Data* (JC/2018/04), 15 March 2018 (https://www.esma.europa.eu/sites/default/files/library/jc-2018-04_joint_committee_final_report_on_big_data.pdf); Basel Committee on Banking Supervision (BCBS), *Sound Practices: implications of fintech developments for banks and bank supervisors*, February 2018 (https://www.bis.org/bcbs/publ/d431.pdf); Caruana (2016), Arner et al. (2017), and Broeders and Prenio (2018).

^8^Source: KPMG, *The Pulse of Fintech 2018*, 2019.

^9^See Slaughter and May (2017).

^10^See Institute of International Finance (IIF), *Deploying RegTech Against Financial Crime*, Report of the IIF RegTech Working Group, March 2017 (https://www.iif.com/portals/0/Files/private/32370132_aml_final_id.pdf); van Liebergen (2017).

^11^See Financial Stability Board (FSB) (2017b) and BaFin (2018).

^12^See Kuroda (2017).

^13^On this topic see Athey (2017), Lepri et al. (2017), and Randell (2018).

^14^See FRA – European Union Agency For Fundamental Rights, *#BigData: Discrimination in data-supported decision making*, May 2018 (https://fra.europa.eu/sites/default/files/fra_uploads/fra-2018-focus-big-data_en.pdf); World Economic Forum, *How to Prevent Discriminatory Outcomes in Machine Learning*, 12 March 2018 (http://www3.weforum.org/docs/WEF_40065_White_Paper_How_to_Prevent_Discriminatory_Outcomes_in_Machine_Learning.pdf); O'Neil (2016)and Žliobaitė (2017).

^15^See Pasquale (2015), Knight (2017), Goodman and Flaxman (2017), Olhede and Rodrigues (2017), and Wachter et al. (2017).

^16^See Koh and Liang (2017).

^17^On this topic see Kroll et al. (2017) and Latonero (2018); Council of Europe, Committee of experts on internet intermediaries (MSI-NET), *Algorithms and human rights - Study on the human rights dimensions of automated data processing techniques and possible regulatory implications*, March 2018 (https://edoc.coe.int/en/internet/7589-algorithms-and-human-rights-study-on-the-human-rights-dimensions-of-automated-data-processing-techniques-and-possible-regulatory-implications.html); *European Parliament resolution of 16 February 2017 with recommendations to the Commission on Civil Law Rules on Robotics* (2015/2103(INL); https://eur-lex.europa.eu/legal-content/EN/TXT/PDF/?uri=CELEX:52017IP0051&from=en).

^18^See the *OECD Principles on Artificial Intelligence* adopted on 22 May 2019 by OECD member countries when they approved the *OECD Council Recommendation on Artificial Intelligence* (https://legalinstruments.oecd.org/en/instruments/OECD-LEGAL-0449); European Commission's High-Level Expert Group on Artificial Intelligence (AI HLEG), *Policy and investment recommendations for trustworthy Artificial Intelligence*, June 2019 (https://ec.europa.eu/newsroom/dae/document.cfm?doc_id=60343) and *Ethics Guidelines for Trustworthy Artificial Intelligence*, April 2019 (https://ec.europa.eu/newsroom/dae/document.cfm?doc_id=60419).
